# A Systematic Study of the Limits of Life in Mixed Ion Solutions: Physicochemical Parameters Do Not Predict Habitability

**DOI:** 10.3389/fmicb.2020.01478

**Published:** 2020-06-26

**Authors:** Adam H. Stevens, Charles S. Cockell

**Affiliations:** UK Centre for Astrobiology, School of Physics and Astronomy, The University of Edinburgh, Edinburgh, United Kingdom

**Keywords:** extremophile, salt, physicochemical, habitability, limits, ions

## Abstract

This study investigated what defines the limits of life in mixed ion solutions. Better understanding these limits should allow us to better predict the habitability of extreme environments on the Earth and extraterrestrial environments. We systematically examined the response of *Bacillus subtilis*, a well characterized non-halophile model organism, to a range of solutions made from single and mixed salts up to their solubility limits and measured at what concentration growth was arrested, specifically exploring Na, Mg, and Ca cations and Cl, SO_4_, and ClO_4_ anions. We measured the physicochemical properties of the solutions to identify which properties correlated with the limits of growth. Individual salts imposed a growth limit specific to the combination of cation and anion, although we generally observe that chloride salts allow growth at lower water activity than sulfate salts, with perchlorate restricting growth even at the highest measured water activity. Growth was limited at a wide range of ionic strength, with no apparently correlation. Despite the theoretically counteracting disordering effects (chaotropic) of perchlorates and ordering effects (kosmotropic) effects of sulfates, when these salts were combined they instead additively narrowed the window for growth in both the Na and Mg cation systems, in the same manner as the combined effects of two chaotropic Ca salts. Our results imply that away from hard limits that might be imposed by physicochemical properties such as water activity, ionic strength or chaotropicity in highly concentrated brines, these properties do not set the limits of life. Instead these limits are highly specific to the salts and organisms in question. This specificity means that the habitability of extreme environments cannot be predicted, even with accurate measurements of the physicochemical conditions present.

## Introduction

A wide diversity of organisms have been found in highly saline terrestrial environments (Andrei et al., 2017). These organisms have a number of strategies for halotolerance, and some are halophiles that cannot grow below a certain concentration of salt (Bowers and Wiegel, 2011). In general, terrestrial aqueous environments are dominated by sodium and chloride ions, but we now know that elsewhere in the solar system where other ions appear to be dominant (Fox-Powell et al., 2016; Vance et al., 2018), and some aqueous systems dominated by other ions do exist on Earth (Dickson et al., 2013; Pontefract et al., 2017). Understanding what factors determine the habitability of aqueous environments with more than one component therefore has applications to the microbiology of extreme environments on Earth and to our search for life elsewhere in the Solar System.

Previous investigations have identified a putative water activity limit for life (Stevenson et al., 2014) caused by a range of biochemical effects including the thermodynamic unavailability of water molecules (Parsegian, 2002). However, there is evidence that other factors such as ionic strength (Fox-Powell et al., 2016) and chaotropicity/kosmotropicity (Williams and Hallsworth, 2009) also have important effects on life (Chin et al., 2010). These properties are not always directly correlated with each other and depend strongly on the solutes in question. Because of this, there are many aqueous environments with a water activity above the theoretical lower limit for life that are still uninhabitable to many organisms for other reasons. Furthermore, the currently defined limits imposed by physicochemical properties may represent our incomplete knowledge of life rather than fundamental limits to habitability. The majority of known organisms are unable to grow in conditions near these measured limits, but halotolerant or halophilic organisms have evolved adaptations that enable metabolic activity at high concentrations, currently defining limits. Irrespective of these adaptations, we can hypothesize the existence of a theoretical limit for a given physical parameter, caused by thermodynamic requirements rather than metabolic considerations. Whether the limits measured so far align with these putative hard physicochemical limits is unknown.

On Earth, brines do exist with extremely high salinities (Antunes et al., 2015) and some with salts other than NaCl, which tends to be the dominant salt in aqueous environments on the planet. As we continue to explore the Solar System we are finding aqueous environments with conditions and compositions very different from terrestrial brines, including a wide variety of ions such as sulfates and perchlorates that rarely form the bulk of water bodies on Earth (Hecht et al., 2009). These extraterrestrial examples range from large-scale, sustained environments such as the subsurface oceans of the icy moons (Vance et al., 2018) to small-scale, transient aqueous activity such as deliquescence at the martian surface (Martin-Torres et al., 2015) where solutions would be highly concentrated due to high evaporation rates.

Our understanding of how these more exotic ion solutions interact with life is even less well developed than for concentrated NaCl, and the theoretical understanding of multicomponent brines is limited, especially at high ionic strength (Pitzer and Kim, 1993). Practical investigations of exotic salts have generally focused on solutions of a single salt with applications to specific environments (Wilks et al., 2019) or restricted concentrations of diverse brines (Heinz et al., 2019). To investigate which properties of aqueous solutions control their habitability, we tested the response of a single organism to multiple combinations of dissolved salts across wide concentration ranges, allowing us to attempt to identify which relevant aqueous property was the primary driver of changes in habitability. This is the first systematic study of the effects of multi-component brines on microorganisms. Understanding complex ion solutions should allow us to better define the habitability of environments without the use of culture dependent experiments that carry an inherent bias caused by the specific capabilities of particular organisms.

## Materials and Methods

### Organism

*Bacillus subtilis* (strain DSM 10T) was used because it is a well-characterized and studied model organism found in soils and the digestive tracts of animals. We also exploit the fact that since the natural habitat of *B. subtilis* is regularly subject to changing osmolarity, it is generally resistant to the rapid changes required by our brine experiments (Hahne et al., 2010) but does not introduce the same bias of adaptations to particular salts carried by excessively halotolerant or halophile organisms. Although there have been a number of studies examining how *B. subtilis* responds to osmotic stress (Völker et al., 1992; Kunst and Rapoport, 1995; Ikeuchi et al., 2003), and it has been shown to increase halotolerance in crops when added to soils (Zhang et al., 2014), our study appears to be the first measurement of the organisms’ response to exotic salts.

### Growth Experiments

Solutions were made by mixing one or two pure salts. The ions investigated were sodium, magnesium and calcium cations and chloride, sulfate and perchlorate anions. These were chosen to correspond to measurements and modeling of ions known to be present at the martian surface (Hecht et al., 2009; Tosca et al., 2011; Glavin et al., 2013). Calcium sulfate is highly insoluble and was not used. Each solution was initially mixed at a range of molar concentrations up to saturation using milli-Q water. Further experiments used these initial results to narrow the range of concentrations across the upper limit of growth, enabling us to build up a large database of measurements for each salt that identify the limits of growth. Combinations of salts were chosen to investigate how mixing two kosmotropic and chaotropic salts – Na_2_SO_4_ (kosmotropic) and NaClO_4_ (chaotropic), and MgSO_4_ (kosmotropic) and Mg(ClO_4_)_2_ (chaotropic) – compares to the effects of mixing two chaotropic salts [CaCl_2_ and Ca(ClO_4_)_2_].

To find growth limits, 190 μl of each ion solution was mixed in 96-well plate with 10 μl of 10 g l^–1^ yeast extract and 10 g l^–1^ casamino acid solution to prevent nutrient limitation being a factor in the lack of growth (Gray et al., 2019). Each well was inoculated with 10 μl of an overnight culture of *Bacillus subtilis.* An OD_600_ (optical density at 600 nm) growth curve for each well was measured in a BMG SPECTROStar Nano plate reader over 96 h at 20°C. Positive and negative controls were included on each plate. Negative controls were either 190 μl of water or LB media with no inoculant and positive controls were 10 μl of overnight culture in either 190 μl of water or LB media without additional salts. Growth or lack of growth was determined by the presence of a standard lag/expontential/stationary growth curve of greater than OD 0.1, and confirmed using an endpoint OD_600_ well-scan of each plate (25 × 25 pixels per well) to determine if visible microbial growth was present. We also defined an intermediate growth condition where growth was visible, but a standard growth curve was not observed. After confirming the growth limit with three independent runs, triplicate wells were plated out onto Nutrient Agar plates to determine if the *Bacillus subtilis* had been killed by the brines or merely prevented from growing.

### Physicochemical Measurements

Physicochemical properties of the brine were measured at the ranges of concentrations of different salts used, at 20°C. Water activity was measured with a Rotronic HC2-AW probe which was calibrated using saturated single salt solutions with known water activity (Winston and Bates, 1960). Water activity for each brine was interpolated across the full range of measurements using a 2nd order quadratic fit.

Ionic strength was calculated using the relation:

$$I=\frac{1}{2}\,\sum m_{j}\,Z_{j}^{2}$$

where *m* is the molarity of a given cation or anion, *z* is the charge of a given cation or anion, and the sum is over all cations and anions (*j)* in the mixture. In mixed brines, water activity and ionic strength were interpolated in two dimensions using 2nd and 1st order quadratic fits, respectively, in Matlab’s curve fitting toolbox.

The chaotropicity or kosmotropicity of a solution is more difficult to measure than its water activity or ionic strength. Cray et al. (2013) proposed a quantitative measure of these properties that form a continuous scale, but the laboratory measurement is difficult to make for highly chaotropic solutes such as perchlorates, is dependent on environmental conditions, and existing data are limited. There are suggestions that chaotropicity and kosmotropicity are linked to the Hofmeister series, and can therefore be inferred relatively from the series, but the basis of this link is not fully understood and the measurements of Cray et al. (2013) do not correspond directly with Hofmeister ranking of cations (Marcus, 2012). Available data for the salts used in this study are shown in Table 1, in ranked order (Cray et al., 2013). Quantitative measurements were not available for perchlorate salts, but they are known to be strongly chaotropic (Asciutto et al., 2010). No data is currently available for how combinations of these salts influences the total chaotropicity or kosmotropicity. We calculated the chaotropic activity for the salts at specific concentrations with available data using the values of Cray et al. (2013).

**TABLE 1:** Chaotropic activity of the salts used in this study, from Cray et al. (2013).

**Salt**	**Chaotropic (+) or kosmotropic (−) activity / kJ kg^–1^ mole^–1^**
NaClO_4_	No data
Mg(ClO_4_)_2_	No data
Ca(ClO_4_)_2_	No data
CaCl_2_	+92.2
MgCl_2_	+54.0
NaCl	−11.0
Na_2_SO_4_	−50.5
MgSO_4_	−64.5

**No data is available for perchlorate salts.**

## Results

**FIGURE 1:**
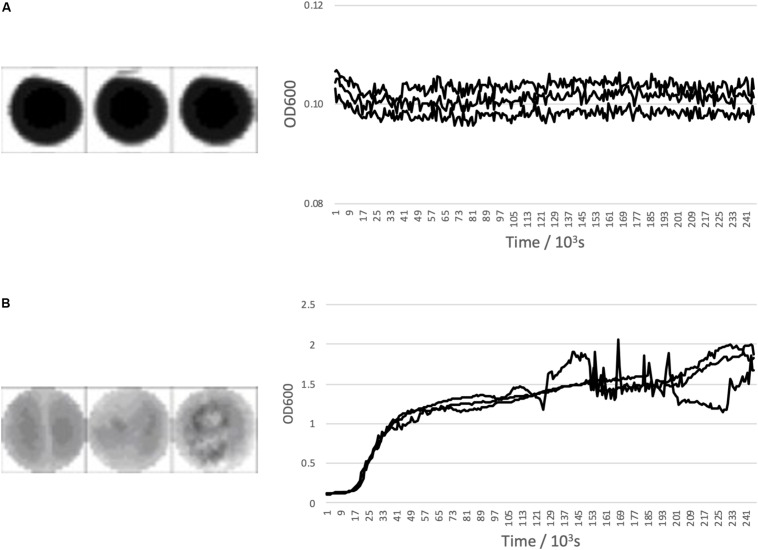
*Bacillus subtilis* growth curve controls. **(A)** Shows negative control triplicate well scans and growth curves and **(B)** Shows a positive control triplicate well scans and growth curves with *Bacillus subtilis* inoculated media. All are across 96 h.

Figure 1 shows a representative examples of expected growth curves obtained with negative and positive controls as observed in well-scan and growth curve measurement modes.

### Individual Brines

**FIGURE 2:**
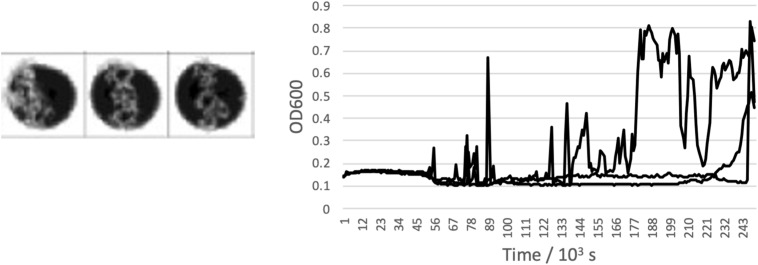
Example of an “intermediate” growth condition observed in CaCl_2_ brine. Clumping means that while growth is visible in the well scan, standard growth curves were not measured in any of the triplicates wells.

The highest concentrations where growth was measured in brines of each of the salts are shown in Table 2, alongside the measured water activity and calculated ionic strength at this concentration. In some cases, the growth limit was a sharp cutoff, but in others we observed a gradual change from a standard growth curve to no growth, with behavior including extended lag phase and extensive clumping that did not produce a standard growth curve (Figure 2). The concentration range of this intermediate growth condition is reflected in the uncertainty quoted in Table 2 – a smaller uncertainty implies a sharper growth cutoff and a larger uncertainty implies a wider range of intermediate growth. Sodium and magnesium sulfate are highlighted because growth was observed up to within errors of the saturation point.

**TABLE 2:** Highest concentration of single salt solutions where growth was measured, along with the measured water activity and calculated ionic strength and chaotropic activity at these concentrations.

	**Highest growth concentration/mol l^–1^**	**Water activity at limit ± 0.002**	**Molar ionic strength at limit/mol l^–1^**	**Chaotropic activity at limit/kJ kg^–1^**
NaClO_4_	0.77 ± 0.04	0.974	0.77	
Mg(ClO_4_)_2_	0.60 ± 0.05	0.975	3.60	
Ca(ClO_4_)_2_	0.30 ± 0.05	0.986	0.90	
CaCl_2_	0.80 ± 0.10	0.960	2.10	73.76
MgCl_2_	0.90 ± 0.10	0.948	2.70	48.6
NaCl	2.20 ± 0.20	0.929	2.30	–24.2
*Na_2_SO_4_*	*0.85 ± 0.05*	0.972	2.55	–42.9
*MgSO*_4_	*1.80 ± 0.10*	0.959	7.20	–116.1

**Na_2_SO_4_ and MgSO_4_ are highlighted because growth was observed up to within errors of the saturation point.**

**FIGURE 3:**
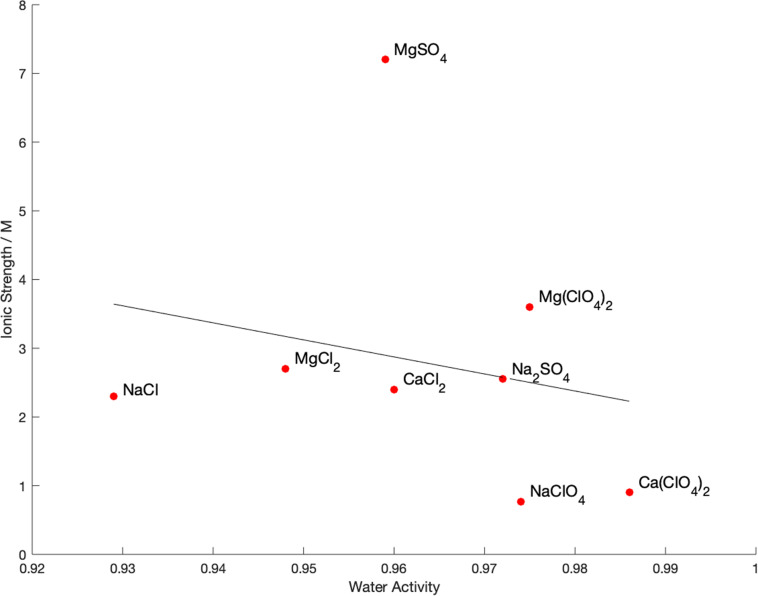
Measured physicochemical properties of brines at the limit of growth of *Bacillus subtilis* in single-salt brines. A least-squares fit line is shown for illustrative purposes, but *R*^2^ = 0.05 so there is no correlation between these values.

The water activity and ionic strength of each of the brines at the limit of growth is compared in Figure 3. There is no apparent correlation (*R*^2^ = 0.05) between the water activity and ionic strength at the limits of growth, nor a particular cutoff value of either property. This is also true of the calculated chaotropic activity, which showed a wide range of values with no correlation to the growth limits. The only observable pattern is that, in general, chloride salts permitted growth at lower water activity than sulfate salts, with perchlorate restricting growth at higher water activities than the other salts.

When plated out after growth experiments, the affected microbes were able to grow again in some cases, but not in others. In particular, the perchlorate solutions either killed or destroyed the cells when above the concentration where growth was limited and did not regrow when played after treatment, whereas in the chloride and sulfate solutions the *Bacillus subtilis* was able to continue growing on a plate after being removed from the salt solutions.

### Mixed Salts

**FIGURE 4:**
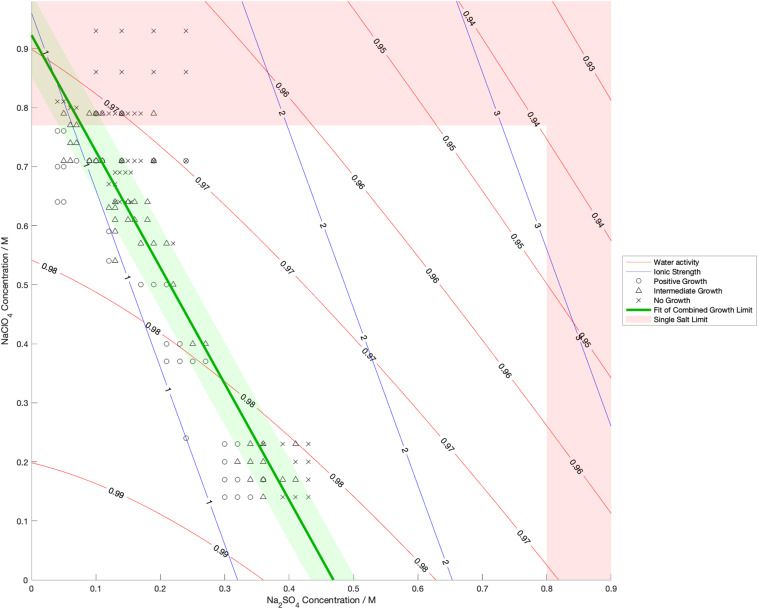
Limits of growth in mixed Na_2_SO_4_-NaClO_4_ brines at varying concentrations. Measured water activity and calculated ionic strength are shown as contours of constant value. All collected growth data are show in gray, with a best fit line and 1 standard deviation error shown in green.

**FIGURE 5:**
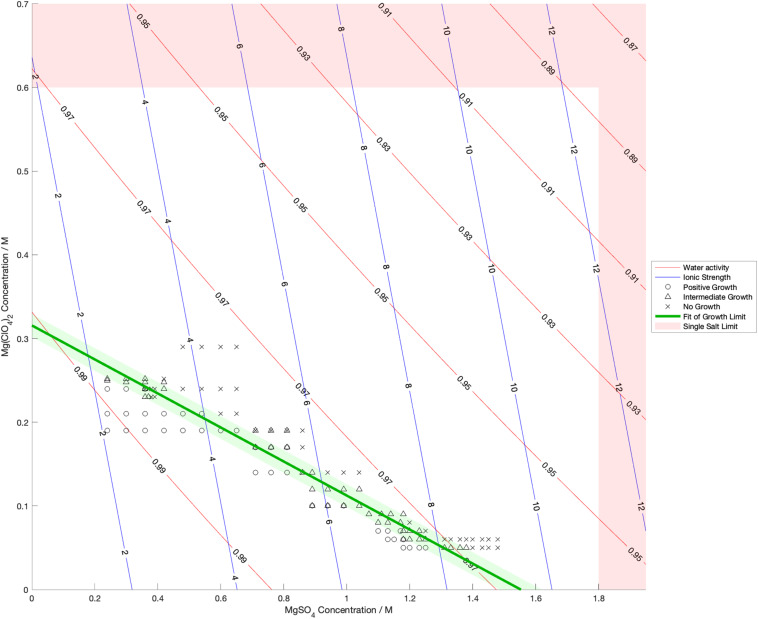
Limits of growth in mixed MgSO_4_-Mg(ClO_4_)_2_ brines at varying concentrations. Measured water activity and calculated ionic strength are shown as contours of constant value. All collected growth data are shown in gray, with a best fit line and 1 standard deviation error shown in green.

**FIGURE 6:**
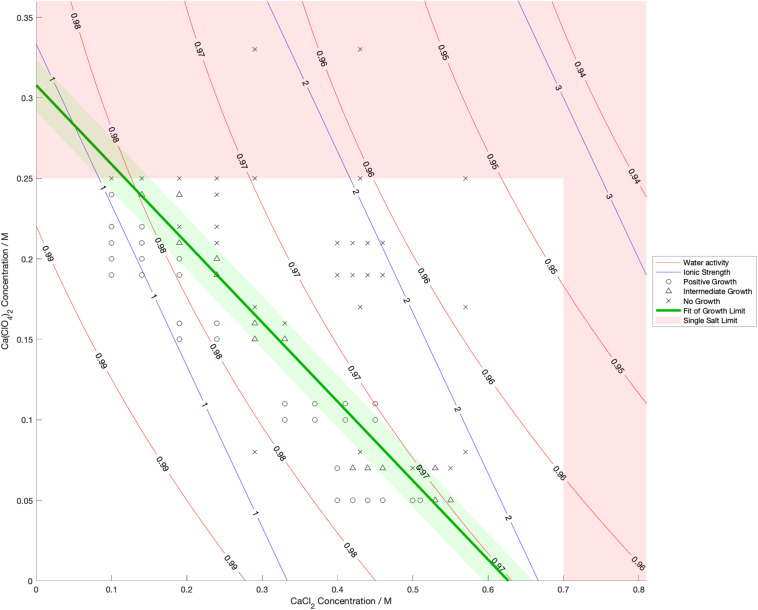
Limits of growth in mixed CaCl_2_-Ca(ClO_4_)_2_ brines at varying concentrations. Measured water activity and calculated ionic strength are shown as contours of constant value. All collected growth data are shown in gray, with a best fit line and 1 standard deviation error shown in green.

Water activity was measured and ionic strength was calculated for a range of mixed salt solutions, producing 2D surfaces of these properties. The measured limits of growth in the mixed brines are overlaid on these surfaces in Figures 4–6, along with the growth limits for solutions of the salts on their own.

## Discussion

We systematically investigated the effects of mixed ion solutions on the growth of *Bacillus subtilis*. This allowed us to measure the response of a single organism to multiple combinations of dissolved salts and determine if the physicochemical properties of the solutions control habitability.

Our results show that neither water activity or ionic strength alone control the habitability of ionic solutions. If they did, we would expect to see correlation along one or both axes in Figure 3. Instead, there is no cutoff at a particular value of water activity or ionic strength and the habitability of each solution is independent of these values. In addition, there appears to be no correlation with specific groupings of cations or anions. Each combination of cation and ion (salt) dictates its own growth limit, although there are some general trends, such as perchlorate being more detrimental to growth than the other anions, which agrees with other studies (Heinz et al., 2019).

This lack of influence does not contradict studies that have shown halophilic organisms to be limited in environments with low water activity or high ionic strength (Stevenson et al., 2014; Fox-Powell et al., 2016), but instead shows that away from thermodynamic limits, individual physicochemical properties or simple combinations of properties are not the main controls on habitability. Instead, our study strengthens previous results that show the biochemical effects of ions in solution are highly specific to the ions, the organisms and any biological macromolecules under study (Warren and Cheatum, 1966; Stevens et al., 2019).

We also found that the disordering (chaotropic) or ordering (kosmotropic) effects of ions cannot explain our results. If particular cations or anions were controlling habitability via their chaotropic or kosmotropic effects, we would expect that a combination of chaotropic and kosmotropic salts in solution would be more habitable than solutions of a single chaotropic or kosmotropic salt, since the effects would counteract each other. However, the converse is true (Figures 4–6). Mixing chaotropic and kosmotropic salts (Na_2_SO_4_ & NaClO_4_ and MgSO_4_ & Mg(ClO_4_)_2_ – Figures 4, 5) reduced the habitability compared to individual salts at the same concentration, showing that either of these properties is not the main control on habitability away from a hard limit that might be created by high concentrations of chaotropes or kosmotropes. This behavior was the same as when combining two chaotropic salts – CaCl_2_ and Ca(ClO_4_)_2_.

A notable point in these results (Figures 4, 5) is that when small a concentration of NaClO_4_ is mixed with Na_2_SO_4_ and when a small concentration of MgSO_4_ is mixed with Mg(ClO_4_)_2_, the limit of growth does not converge with those of solutions of only Na_2_SO_4_ or Mg(ClO_4_)_2_ respectively. NaClO_4_ and MgSO_4_ appear to cause an asymptotic relationship at low concentrations, reducing the highest concentration of the other salts that allow growth by ∼50% even at < 0.1 M concentration. All other mixed solutions tested, i.e., small amounts of Na_2_SO_4_ in NaClO_4_, small amounts of Mg(ClO_4_)_2_ in MgSO_4_, small amounts of CaCl_2_ in Ca(ClO_4_)_2_, and small amounts of Ca(ClO4)_2_ in CaCl_2_, all converge at the growth limits for the single salts to within experimental errors. This perhaps suggests that NaClO_4_ and MgSO_4_ behave fundamentally differently, at least when in mixed solutions with other salts. These results were highly repeatable, although measuring growth limits at these low concentrations in small volumes of solution means there are large relatively errors. One possible explanation is that the imbalance of monovalent ions in these solutions creates localized charge density effects in the solution that are deleterious to cells, but further study would be required to confirm this and testing further combinations may offer additional insight. In natural aqueous environments with mixed geochemical inputs, small amounts of particular salts could therefore have dramatic effects on habitability, for example if small concentrations of MgSO_4_ were to dissolve in perchlorate-rich surface waters formed by deliquescence on Mars.

The uncertainty in how physical properties affect habitability is complicated by the fact some salts showed a relatively sharp cut-off in habitability at a particular concentration and in others the transition from habitable to uninhabitable conditions was less well defined. These intermediate growth states imply that the solutions are causing changes in metabolic responses, perhaps by extending the lag phase, for example because more energy is required for metabolic maintenance, or by inducing biofilm formation or clumping as we observed, all of which are known growth strategies for *Bacillus subtilis* under extreme conditions (Gray et al., 2019). Future investigations using different organisms could give insight into whether these responses are common across species, classes and domains, or if each organism responds differently.

We should note that we have used the definition of habitability as being when an organism can grow and reproduce in an environment, rather than the broader definition that includes an organism simply maintaining its own metabolism or remaining dormant. While our experiments showed the limits of growth in ionic solutions, in some cases the organisms were able to grow after being removed from the solutions, suggesting they were inactive but still viable, whereas in some solutions (notably perchlorate) there was no growth even after removal from the solutions, suggesting the cells had been killed. Even though this implies deactivation or spore formation of the *Bacillus subtilis* cells in some solutions, rather than their death, the lack of growth and reproduction under these conditions is the critical point for habitability because it means propagation cannot occur. While the cells may germinate again in less extreme conditions, the solution itself remains uninhabitable, and inactive organisms would eventually lose viability over sufficient timescales.

Interactions of these physicochemical properties with other thermodynamic properties also adds complexity to the assessment of habitability. Studies have shown that salts can enhance, rather than diminish, the survival of microorganisms at low temperature (Heinz et al., 2018) and specifically chaotropic solutes have been shown to counteract the rigidification of biomolecules due to low temperatures (Ball and Hallsworth, 2015).

Martian aqueous systems appear to have been far more complicated than the NaCl dominated systems of Earth (Tosca et al., 2011), with relatively high levels of perchlorate ions and divalent ions like magnesium, calcium and sulfate (Kounaves et al., 2010, 2014), and will have changed significantly over Mars’ history as the surface dried out and the geochemistry changed (Rapin et al., 2019). Our data confirm previous results that show perchlorate is generally more deleterious to life in the environments, but also suggest that there are sensitive differences between perchlorate salts and that magnesium and calcium perchlorate salts, which appear to be more common on Mars (Kounaves et al., 2014) limit habitability less than sodium perchlorate when in mixed solutions. Early in Mars’ history, before aqueous environments became so concentrated that hard limits of properties like water activity became dominant (Tosca et al., 2008) the habitability of different environments would have been highly variable and dependent on the specific geochemical history because of the ion-specific habitability effects we have identified.

If habitability is controlled by a complex combination of environmental properties, it may not be possible to robustly predict the habitability of an unknown aqueous environment, even if we were able to measure its physicochemical properties directly. This aligns with previous findings showing that the effects of solutes on particular macromolecules are highly specific and hard to predict (Ball and Hallsworth, 2015). Ultimately, this implies that habitability can only truly be tested by inoculating a specific environment with a specific organism, and such a test will only be valid for that organism (Cockell et al., 2019). We are left with a view of habitability in ionic solutions that is unpredictable except at the edges of life where limits are imposed by fundamental thermodynamic considerations. Away from these hard limits the activity of organisms is sensitively defined by the specific combination of ions and the specific adaptations of the organism itself. Any transient surface brines or subsurface brines present on Mars today will be highly concentrated with salts and may be habitable to halophilic terrestrial life or native organisms that were able to adapt to the increasing concentration of salts, but we cannot predict this directly from measurements of the brine alone.

## Conclusion

These conclusions have applications to the issue of planetary protection. They imply that habitability measurements are not generalizable to the full range of potentially contaminating organisms that may be transported to other planets. Our data suggest two possibilities. Either the interacting effects of ions on microorganisms are more complex than the direct effects of parameters such as water activity, ionic strength and chaotropicity and that more investigations will improve our predictive capacity, or ion effects are highly sensitive to the specific metabolic and physiological characteristics of given organisms and are therefore so highly specific they cannot be *a priori* predictable. Thus, habitability is not an objective criterion under these circumstances, but can only be defined against a given organism, except at the extreme edges where limits are imposed by fundamental thermodynamic considerations. Away from these hard limits, further investigations are required to fully understand how habitability is affected by combinations of different ions in solution and how generalizable these results are to other organisms with varied metabolisms.

## Data Availability Statement

All datasets generated for this study are included in the article/Supplementary Material.

## Author Contributions

AS and CC designed the study and experiments and wrote the manuscript. AS collected and analyzed the data. Both authors contributed to the article and approved the submitted version.

## Conflict of Interest

The authors declare that the research was conducted in the absence of any commercial or financial relationships that could be construed as a potential conflict of interest.
